# The Incidence of Plague on Different Classes

**Published:** 1898-01-08

**Authors:** 


### THE INCIDENCE OF PLAGUE ON DIFFERENT CLASSES.

It is becoming more and more apparent that the outbreaks of plague which have occurred in Bombay and in other Indian towns are but a climax to a gradually increasing deterioration of health conditions which has been going on for some time. That overcrowding and lack of ventilation are at the root of much of the mischief is strongly suggested by certain facts which have been ascertained in regard to the incidence of the disease in the different quarters of Bombay. In Kamatipura, one of the most unhealthy areas of the native town, the municipal halakores and sweepers, in daily contact with the filth of infected houses, are said to have escaped the disease, though living in chawls similar to those in which people were dying by hundreds. The women of a disreputable class who lived in that quarter also largely escaped the plague. These interesting facts have been investigated by Dr. Rogers Pasha and Dr. Bitter, who, after visiting the sweepers' quarters and the localities in which the women dwelt, attributed their immunity to the ample ventilation and the absence of overcrowding in their dwellings. The immunity of these two classes, to which attention is drawn in the report of the municipal health officer, seems to point strongly to some of the insanitary conditions which are operative in causing a prevalence of plague. Not that we would for a moment suggest that the problem of the etiology of plague is a simple one. Indeed, as showing how complex are the conditions to be studied, we would refer to what is now greatly puzzling people in Bombay, viz., the comparatively heavy incidence of plague during the last few months upon the Jains. The Jains are a comparatively well-to-do community, reputed to be of cleanly habits, yet they have recently been suffering from plague in far higher proportion than the rest of the inhabitants of the city. The varied incidence of plague upon the different communities within a city is a subject which will repay the most careful study, especially where each class is dominated and tied down by custom to the extent that still holds good in India.

